# Beyond the borders: the gates and fences of neuroimmune interaction

**DOI:** 10.3389/fnint.2014.00026

**Published:** 2014-03-13

**Authors:** Javier Velázquez-Moctezuma, Emilio Domínguez-Salazar, Beatriz Gómez-González

**Affiliations:** Area of Neurosciences, Biology of Reproduction Department, CBS, Universidad Autónoma Metropolitana, Unidad IztapalapaMexico City, Mexico

**Keywords:** neuroimmunomodulation, neuroimmunology, neuroendocrinology, brain barriers, neuroimmunoendocrinology

Historically, in most organisms the nervous, immune, and the endocrine systems have been studied as independent components. However, during the last decades, growing evidence supports the notion that these are three parts of a unique system, the neuro-immune-endocrine system (Besedosky and Rey, 2007). Both clinical observations and experimental data obtained from animals reveal a close relationship among the three components of the system. This is the theme of this Research Topic.

The literature contains a large number of reports concerning the relationship between two of the three components: neuro-immune, neuro-endocrine, and immune-endocrine. More recently, the third component of the triad has been added to the study of stress (Baumann and Turpin, 2010) and depression (Hernández et al., 2013). A similar situation has been reported for neuro-immune mechanisms in which an endocrine component is now disclosed, e.g., irritable bowel syndrome (Stasi et al., 2012). Thus, if we consider that the neuro-immune-endocrine system is just one complex regulatory system, then the understanding of the interactions among the three systems can lead us to analyze many pathological states, which have usually been studied as a single disequilibrium of one of these three components, such as rheumathoid arthritis (del Rey et al., 2010), and depression (Hernández et al., 2013).

As part of their independent study, the three systems were characterized as having highly specialized signaling molecules that constituted a fence against mutual interaction; neurotransmitters were described for neural communication, hormones for endocrine communication and cytokines and chemokines for immune signaling. However, as the characterization of both the signaling molecules and the receptor systems progressed, the fences transformed into gates for direct neuro-immune-endocrine communication; receptors for neural derived signals were found in both the endocrine and immune systems. Cytokine production was described inside the central nervous system and the hormones were shown to signal both the neural and immune cells. The only fences left were the barriers precluding direct contact between the cellular and molecular components of the three systems, particularly the brain barriers. Those barriers were shown to have localized fences that allowed selective interaction among the cellular and molecular components of the three systems in a highly regulated manner.

This Research Topic includes original reports, reviews and minireviews regarding the description of the gates and fences in neuro-immune-endocrine interactions. It contains four sections; in the first section three papers describe the gates and fences for neuroimmune interactions directly at the central nervous system. Stolp et al. (2013) describe the changes in neuro-immune interactions through the brain barriers during early development and ageing. Hurtado-Alvarado et al. (2013) present evidence on the role of pericytes, a blood-brain barrier cellular component, in the regulation of the immune response in the brain under both physiological and pathological conditions. Chavarría and Cárdenas (2013) review the influence of neurons and glial cells on the immune response once immune cells have trespassed the brain barriers, molecules promoting an immuno-modulatory environment in the brain are described.

The second section includes two reviews emphasizing the role of hormones on neuro-immune interactions. Quintanar and Guzmán-Soto (2013) describe the role of hypothalamic neuro-hormones in peripheral immune responses, including the clinical relevance of those hormones. Monasterio et al. (2013) discuss the participation of prolactin and progesterone in the regulation of immune responses in the central nervous system of pregnant and lactating females.

The third section contains three papers that describe interactions between the brain and gut. Montiel-Castro et al. (2013) describe the role of the immune system in the cross-talk between the gut microbiota and the brain, focusing in the description of the regulatory effects of microbiota on brain physiology and behavior. Campos-Rodríguez et al. (2013) present evidence regarding the role of stress hormones on the intestinal immune response, both at the cellular and molecular levels. Garzoni et al. (2013) review the neuro-immune mechanisms mediating the development of antenatal intestinal inflammatory response, with special emphasis on the role of the cholinergic anti-inflammatory pathway in the generation of necrotizing enterocolitis.

Finally, the fourth section includes two papers discussing the alteration in neuro-immuno-endocrine interactions in disease. The paper by Meraz-Ríos et al. (2013) discusses the role of inflammatory signals in the exacerbation of the hallmark pathophysiological changes in Alzheimer's disease. In addition, they also discuss the outcomes of the use of anti-inflammatory drugs and immunotherapy to prevent and/or reduce neuroinflammation in patients suffering Alzheimer's disease. Finally, León-Cabrera et al. (2013) describe the relationship among leptin levels, inflammatory mediators and metabolic changes in the Mexican population, aiming to establish a profile of neuro-immune-endocrine factors ensuing the generation of metabolic syndrome.

All the papers included in this volume are just a sample of the large amount of research that should be done in the forward years to understand the mechanisms underlying the gates and fences of neuro-immuno-endocrine interactions. As editors, we would like to express our gratitude to all the scientists that collaborated to finally get this volume, both authors and reviewers; the effort and careful work of all of them undoubtedly led to the high academic value of this volume.
